# Supporting parent treatment decision-making in relapsed and refractory neuroblastoma: co-design of a web-based intervention

**DOI:** 10.1186/s12911-025-03313-z

**Published:** 2025-12-08

**Authors:** Helen Pearson, Anne-Sophie Darlington, Faith Gibson, Michelle Myall

**Affiliations:** 1 School of Health Sciences, University of Southampton, Southampton, UK; 2 The Oak Centre for Children and Young People, The Royal Marsden NHS Foundation Trust, Sutton, Surrey UK; 3 Centre for Outcomes and Experience Research in Children’s Health, Illness and Disability (ORCHID), Great Ormond Street Hospital for Children NHS Foundation Trust, London, UK; 4 School of Health Sciences, University of Surrey, Guildford, Surrey UK

**Keywords:** Decision-making, Childhood cancer, Co-design, Intervention development, Medical Research Council, Psycho-oncology, Qualitative

## Abstract

**Background:**

Parents of children diagnosed with relapsed or refractory neuroblastoma become involved in making treatment decisions for their child due to an absence of no standard treatment protocol with no clear treatment endpoints. Relapsed and refractory neuroblastoma is a poor-prognosis childhood cancer with varying treatment options available depending on their child’s response to treatment. As a result, parents in partnership with their child’s medical team make repeated treatment decisions over time. Research has shown how this decision-making is influenced by uncertainty of their child’s response to treatments and overall outcome, and parents’ emotional and cognitive adjustments. Having time to research and gather information has also shown to enable and inform parent involvement and responsibility within decision-making. An intervention to support parents can help them navigate these complex decisions aiding their cognitive, emotional, and practical needs to enable and inform their decision-making.

**Methods:**

Intervention development followed the Medical Research Council Framework for developing complex intervention co-designed with a parent stakeholder group. A review of the literature and analysis of parent interviews informed the intervention. A one-off clinical advisory group was formed to review draft content. Intervention user testing was completed using cognitive think-aloud interviews.

**Results:**

A web-based intervention was developed to support and facilitate parent treatment decision-making in relapsed and refractory neuroblastoma. Co-design was iterative with a combination of ten face-to-face and virtual workshops to discuss and develop the website content, design, and layout. User testing was completed with seven parents and findings informed changes which included reformatting web pages, reducing text paragraphs for easier reading, creating additional webpages for ease of navigation of information and providing parent quotes for authenticity. Recommendations for intervention development using co-design are provided based on our experiences of using this approach.

**Conclusions:**

This is a disease specific intervention developed to support and facilitate parent treatment decision-making in a specific poor-prognosis childhood cancer. Co-design was essential to ensure the intervention met the needs of this parent population. Further work following the MRC framework will test and evaluate its impact and effectiveness in clinical practice.

**Supplementary Information:**

The online version contains supplementary material available at 10.1186/s12911-025-03313-z.

## Background

Neuroblastoma is a childhood cancer predominately seen in children under five years of age and accounts for 6% of childhood cancers in the United Kingdom (UK) with approximately 100 children diagnosed each year [1]. Fifty of these children will have high-risk disease with 40 experiencing relapsed or refractory disease associated with poor outcomes [2, 3]. Parents of children diagnosed with relapsed or refractory neuroblastoma become involved in making treatment decisions for their child in partnership with their child’s healthcare team (e.g. medical consultant, clinical nurse specialist, psychologists) as there is no standard treatment protocol with no clear treatment endpoints and various treatment options available. As a result, parents often make repeated treatment decisions depending on their child’s response to treatment, availability of treatments and their child’s clinical condition. Research within paediatric oncology has shown parents want to be involved in making these decisions [4] with parents typically becoming involved when there is no standard of care treatment available [5]. Repeated decision-making in this context can continue for months or years in the hope of their child’s survival. Decisional conflict and regret related to treatment decisions can occur [5–7] which suggest parents need additional support when making treatment decisions for their child. Previous paediatric oncology research has highlighted the importance of developing interventions to support parental decision-making [8] however despite this there continues to be a lack of interventions which support parents in doing so.

The NHS Long-Term Plan [9] placed a focus on digitally enabled care with technology to support patient self-management and involvement in decisions about their care. Within children and young people’s cancer, to encourage involvement a decision aid was developed for parents and adolescents who are considering participation in a clinical trial [10]. This is focused on decision-making for discrete decisions centred on an individual’s values and preferences at the time of making a decision. Frameworks of shared decision-making (SDM) in paediatrics have acknowledged that values and preferences are not stable and change in relation to parent capacity and reflection [11] which could be related to their child’s clinical status, tolerance of treatment and uncertainty of their child’s outcome. This suggests for parents who are making repeated treatment decisions they require an intervention to support their decision-making throughout their child’s treatment which can last for months or years depending on factors as mentioned above.

Our literature review exploring parent values and preferences in treatment decision-making when their child has a poor-prognosis cancer showed the complexity of decision-making extends beyond values and preferences, acknowledging decisions can be heavily influenced by emotions and descriptive processes such as intuition, previous knowledge, and experience [12]. For parents making repeated treatment decisions, an intervention in the form of a decision support tool could facilitate this complex decision-making allowing for a more flexible approach incorporating various concepts which are known to influence, inform and enable parents in making decisions. This intervention once implemented in clinical practice could be evaluated to ascertain whether it has provided practical and emotional support to parents in making these complex decisions.

This work forms part of a wider study exploring parent treatment decision-making in relapsed and refractory neuroblastoma. Through qualitative interviews with parents, the study explored how they made treatment decisions and identified factors which informed, influenced, and enabled parents in their treatment decision-making [13]. Findings showed the first treatment decision was made by their child’s medical consultant. Over time parents entered a collaborative, SDM partnership with their child’s medical consultant, informed by their lived experience and knowledge. However, if treatment options became exhausted in the UK, parents became independent in their decision-making, seeking second opinions, contacting medical consultants internationally and in some cases travelling abroad for clinical trials and experimental therapies. This independent decision-making was influenced by uncertainty and parent cognitive and emotional adjustment wanting to pursue further treatments to have more time with their child. This breakdown in a SDM approach highlighted the need for parents to have additional supporting their decision-making. The study showed the potential of decision support interventions to assist and empower parents to participate fully in making treatment decisions for their child whilst also having opportunity to facilitate SDM between parents and healthcare professionals.

There is a clear need for supporting parents in making complex treatment decisions and therefore this work developed an intervention specifically for this parent group. The aim of this paper is to describe the development process and user testing of a novel intervention for parents which supports their treatment decision-making and conversations with healthcare professionals in the context of treatment choices when their child has relapsed or refractory neuroblastoma. Two research questions informed this work: (1) what intervention delivery, format and content will best support parents?; (2) does the intervention have acceptability and usability for parents?

## Methods

Intervention development followed The Medical Research Council (MRC) Framework for developing complex interventions [14, 15] underpinned by the Framework for actions for intervention development [16]. These actions consider concepts such as how to plan the development process, involve stakeholders, review research evidence, incorporate theories and intervention user testing [16]. The intervention was co-designed, working in partnership with parents (stakeholders) who had lived experience of having a child with neuroblastoma. Co-design is a collaborative process between stakeholders and researchers which leverages experience, knowledge, and insights to ensure the needs of the population the intervention is intended for are addressed [17, 18]. Co-design was important given the sensitivity of the intervention subject and the researchers’ commitment to the intervention being designed with parents for parents to ensure content was appropriate and relevant. This was achieved through establishing parent stakeholder and clinical advisory groups, developing stakeholder workshop structures, considering the foundations to incorporate within the intervention, design of the intervention and intervention user testing.

### Establishing a parent stakeholder group

A call for stakeholder participation in developing the intervention was posted on the study social media platforms (X, Facebook, and Instagram) which has a large parent following. The study was presented to Solving Kids’ Cancer UK Parent Involvement Forum with a request for participation. A total of 11 parents contacted the researcher which resulted in seven parents (four mothers and three fathers) initially committing. One father withdrew after the second workshop as their child became unwell. The wider study had a Patient and Public Involvement group (PPI) from which a member joined the stakeholder group to support the alignment of the intervention work within the context of the overall study. The group was facilitated by a clinical nurse providing support to parents which gave the opportunity to raise any concerns relating to the work if they felt unable to do so directly with the researcher. The parent stakeholder group supported addressing the research question ‘what intervention delivery, format and content will best support parents?’ through the intervention development process.

### Stakeholder workshops

At the outset, six two-hour workshops were scheduled over eight months alternating between face-to-face and virtual. This schedule was based on reviewing the intervention development processes in other published work [10, 19, 20]. Workshops were audio-recorded, summarised with action points to be completed prior to the next meeting and shared with the group for sense checking. The group created a partnership working agreement which focused on collaborative working through valuing all views even if these were not shared by everyone, encouraging the sharing of different perspectives and ideas, and take time to listen and learn from each other. A WhatsApp group was set up between parents as a method of support to one another. Parents were paid for their time and expenses (subsistence and travel) as per the suggested NIHR payment rates [21].

An additional four workshops were added during the development process. Two with the intervention development company, and two as the development process took longer than anticipated. In between workshops, iterative feedback between the group was collated with suggestions and feedback refining the intervention prior to user testing. Whilst user testing was being undertaken, the group suggested ideas to support dissemination and implementation of the intervention. Table 1 details the stakeholder workshop schedule with anticipated aims, actual work completed in each workshop and work undertaken between meetings.

**Table 1:** Stakeholder workshop schedule

Workshop, No. of parents, Meeting platform	Anticipated Meeting Aims	Actual Workshop Schedule	Work undertaken between workshop meetings
WS 1 *N* = 7 F2F	Partnership working agreement Discuss workshop aims and objectives Discuss and define what an intervention is in the context of parent treatment decision-making Present qualitative study results which will inform the intervention Discuss study results and explore the components which should inform the intervention	Partnership working agreement Discuss workshop aims and objectives Discuss and define what an intervention is in the context of parent treatment decision-making Present qualitative study results which will inform the intervention Discuss study results and explore the components which should inform the intervention Identified and agreed aims of the intervention	Group: Think about interventions which could support the identified aims Consider types of resources and support which could address each aim
WS 2 *N* = 6 Virtual	Present different intervention types based on known research Discuss types of resources which could address each aim Discuss ideas on what intervention may suit parent needs within decision-making	Present different intervention types based on known research Discussion on the positive and negatives of an app or web-based intervention Discuss ideas on what intervention may suit parent needs within decision-making Discussion on accessibility of intervention considering written, audio and visual formats	Researcher: Review intervention literature Group: Consider types of resources and support which could address each aim Identify topics for inclusion in intervention
WS 3 *N* = 5 F2F	Continued discussion on positive and negative of app or web-based interventions Discuss types of resources which could address each aim	Continued discussion on positive and negative of app or web-based interventions Agreed intervention type: web-based (website) Suggested companies to develop website considered Discuss types of resources which could address each aim Topics identified for inclusion in intervention Identified healthcare professionals who could be videoed on specific topics	Researcher: Contact three website development companies for initial discussion Drafted intervention content for topics identified Approached healthcare professionals to be involved in video content for the website Group: Review intervention content draft Consider resources for inclusion from reputable sources: Charities, NHS
WS 4 *Additional workshop* *N* = 6 Virtual	*Additional workshop with potential website development company who worked with non-profit organisations and had experience of designing a childhood cancer charity website*	Presentation from the company on their process for website development including examples of previous work Opportunity for the group to ask questions this process and how they envisage working with the group	Group: Feedback on email their thoughts and feelings of the development company Website company officially employed for this work
WS 5 *N* = 6 F2F	Discussion on format and design of intervention	Discussed website development company process and timeline for completion of work Reviewed draft intervention content Feedback on healthcare professional involvement with videos for the website Discussed assembling a Clinical Advisory Group to review intervention content	Researcher: Develop a Clinical Advisory Group for feedback and input into content Organise company to produce healthcare professional videos Revise draft intervention content
WS 6 *Additional workshop* *N* = 6 Virtual	*Additional workshop with website development company to undertake Brand Personality Workshop*	Group work to consider brand (website) values and personalities Values identified are used as core tenets of the design and development decisions made towards the website	Group: Brand values (figure X) and brand personalities (figure X) shared with the group Review updated draft intervention content
WS 7 *N* = 6 F2F	Review design of intervention and feedback Invite intervention development company to meeting	Review draft intervention content and identify any missing topics Review feedback from Clinical Advisory Group, discuss what requires inclusion in intervention	Researcher: Revise draft intervention content Group: Review and feedback on revised draft intervention content
WS 8 Virtual	Review intervention final draft Discuss plans for user testing intervention	Review and feedback on wireframes of website Discuss ongoing development plans and timelines	Researcher: Wireframes feedback to development company Healthcare professional videos recorded for website
WS 9 *Additional workshop* *N* = 6 Virtual		Review website designs Feedback on healthcare professional videos	Researcher: Website designs feedback to development company Draft intervention content given to development company Group: Review website draft Review and feedback on healthcare professional videos to edit content as required
WS 10 *Additional workshop* *N* = 6 Virtual		Feedback on website draft Identify areas of content within the website that require refining – remove duplication and add in signposting to other website pages Discuss ongoing development plans and timelines for website including user testing	Researcher: Website feedback to development company Edit website content Group: Review website after content has been edited Iterative feedback as website continued to be developed and refined

Key: F2F = Face-to-Face; N = number; V = virtual WS = workshop

### Clinical advisory group

Healthcare professionals who specialised in neuroblastoma and/or supported parents in treatment decision-making were invited through national organisations and networks to participate in a one-off clinical advisory group meeting. Eleven healthcare professionals (four medical consultants, four nurses, one psychologist, two social workers) from six children’s cancer primary treatment centres in the UK participated. A parent from the stakeholder group attended the meeting to provide context and contribute to discussions as required. The meeting purpose was to review the draft intervention content, provide feedback, identify topics to consider including and discuss ways to disseminate and implement the intervention into clinical practice.

### Foundations for intervention development

The intervention was informed by a literature review of parent treatment decision-making in poor-prognosis childhood cancer [12] and qualitative interviews with parents who were making treatment decisions for their child who had relapsed or refractory neuroblastoma [13]. The literature review showed parents oscillated between hope, fear and uncertainty whilst making decisions which could prolong their child’s life and minimised suffering [12]. They valued being involved in making decisions and having time to make informed decisions which supported their concept of being a good parent [12]. These findings corresponded with the findings from parent interviews however, these concepts were exacerbated further as they made repeated treatment decisions impacting their cognitive and emotional adjustment to the decision-making process [13]. Parents also felt an increased responsibility and involvement as they made repeated treatment decisions gathering information and seeking second opinions due to treatment options becoming limited and at times a breakdown in the SDM approach with their child’s medical consultant [13]. Corbin & Strauss’s [22] illness work theory informed stakeholder thinking of the theoretical constructs of managing their child illness alongside daily living and how this could support development of the intervention. Theory did not shape the intervention format as the stakeholder group felt this may inhibit the flow of the intervention and lead to confusion if contextualised in this way. However, based on concepts from the qualitative interviews, the stakeholder group saw how the theoretical constructs of illness work, such as managing their child illness through information gathering and daily living supporting their child’s quality of life would be addressed within the intervention.

### Intervention design

The stakeholder group worked with a professional web-design company to develop the intervention and followed their design process. The company were selected based on their experience, reputation from previous clients, understanding of the intervention needs and workable timeframes. Four stages of the design process were considered: Learn, Plan, Apply and Nurture. These stages mirror the development processes of other interventions [10, 23]. Nurture related to the ongoing use and development of the website once it is live. For this reason, it was not included in the design process.

### Intervention user testing

User testing addressed the following research question: ‘does the intervention have acceptability and usability for parents?’ Parents with experience of a child with neuroblastoma, and not involved in the development process, were invited to participate through the study social media platforms. User testing with cognitive think-aloud interviews enabled understanding of how information was perceived, interpreted, and identified any problems with the quality, use or understanding of the intervention [24]. As a frequent method for intervention user testing, cognitive interviews encouraged participants to verbalise their thought processes [25]. Interview questions (Additional file 1) were adapted from other cognitive interview schedules [26].

Interviews were conducted virtually with the lead researcher (HP), audio-recorded and carried out in iterative cycles staggering participants to generate and analyse the feedback to support intervention refinement [16, 18]. Between each iterative cycle, the intervention was amended based on participant feedback. Five to nine participants are recommended for think-aloud interviews [27].

## Results

There were five processes integral to the co-design of the intervention to ensure it met the needs of parents to support them in their decision-making: intervention user needs, content development, clinical advisory group input, the design process and user testing and refinement.

### Identifying user needs

Findings from the literature review and qualitative interviews were discussed within the stakeholder group which generated four intervention aims: (1) provide practical resources to support parents in making decisions; (2) acknowledge, validate, and support the cognitive and emotional impact of decision-making; (3) provide resources to facilitate conversations with healthcare professionals; (4) support parents through making repeated treatment decisions over time.

There was a consensus that the intervention should not duplicate information already available, but signpost to reputable resources providing confidence to parents being directed to the same resources for continuity and consistency. Information needed to be balanced or neutral to avoid the potential for parents to feel guilty or pursue options which they were uncomfortable with, for example accessing treatment outside of the NHS or fundraising for international clinical trials. It was important that the intervention felt welcoming and empowering particularly if relationships with healthcare professionals were strained due to treatment decision-making. Parents’ feelings during their initial interaction with the intervention was considered as indicative of how likely they would be to utilise the intervention and engage with the content. For this reason, the use of graphics, images and colours were considered important to provide a welcoming feeling and safe space in a sensitive subject which holds a lot of emotion for parents.

Parents suggested the inclusion of videos by healthcare professionals on topics such as the psychological processes of decision-making, how to work in partnership with healthcare professionals and seeking a second opinion. Similarly, downloadable documents for example ‘questions to ask your child’s consultant’ to facilitate conversations with healthcare professionals, and a glossary of terms to help parents interpret information. To accommodate parent’s changing needs, an informal built-in feedback mechanism was proposed to enable ongoing intervention refinement.

In terms of the format, parents preferred a web-based intervention that was available via multiple platforms including mobile, desktop or tablet. This would maximise engagement and only required the user to insert or click on a web address to access. While an app was also considered, it was rejected as parents would need to download the app to access this, which placed an immediate barrier in terms of ease of accessibility. Furthermore, the financial set up costs were significantly higher than a website.

### Content development

The content development work was completed in parallel with the intervention design process. Content was developed related to the intervention aims considering health literacy needs. Written content considered word choice (i.e.: plain and sensitive language), concise text paragraphs, bullet points and sub-headings. Content headings identified the relevant information for webpages such as emotional support, understanding the cognitive psychological processes of decision-making and practical support in working with their child’s healthcare team. An initial written draft by the researcher (HP) informed the design process for the wireframes providing headings and sub-headings. Wireframes are the interactive functional designs of the website which helped visualise and communicate the user journey throughout the website. A further nine iterations of the content involved input and feedback from the group. The group collated content information such as devising a list of charities which can support parents practically and emotionally, a list of the parent groups on social media and questions to include in the ‘questions to ask your child’s consultant’ section. During this process additional content, for example, information on considering stopping treatment and videos from parents talking about their decision-making experiences was incorporated were which suggested by the group. Not all stakeholder suggestions were actioned. For example, having a set off questions on the homepage with responses filtered to navigate parents to the information they might be looking for was rejected due to some stakeholder concerns that parents may not be able to articulate what they needed due to their emotional state or the risk they could miss information relevant to them.

Once the website had been developed, the group suggested strategies to support dissemination and implementation. Dissemination included presenting the website at the Solving Kids’ Cancer UK Neuroblastoma Parent Symposium and at national healthcare professional meetings such as the Children and Young People’s Cancer Association annual conference and the UK collaborative for Clinical Cancer Research Children’s Neuroblastoma Group. To support implementation within clinical practice, leaflets and stickers with QR codes for the website were made for healthcare professionals to give out to parents. Members of the stakeholder group who were also members of closed forum parent Facebook groups for neuroblastoma shared the website information across these forums to increase parent awareness. The researcher contacted individual charities who support parents of neuroblastoma such as SKC and Neuroblastoma UK to inform them of the website once it was live.

### Clinical advisory group

Feedback from the clinical advisory group focused on including information on psychological support services and providing an overview of the qualitative findings within phase one of the study. Three medical consultants agreed to review and edit the clinical trials and resources information which was completed outside of the group meeting. In doing so they suggested adding in information and examples of how treatments have developed through clinical trials. This information was drafted by one of the medical consultants. It was also important to consider geographical and regional differences regarding accessing local services, language was amended to reflect this, for example regarding hospice and psychological support. Suggestions for dissemination and implementation were the same as those suggested by the parent stakeholder group. The only additional suggestion was individualising the QR codes on the stickers and leaflets for each children’s cancer treatment centre and add monitoring within the website. This meant we would be able to see which centres are distributing the leaflets and stickers and provide additional support and information to centres who are not using them.

### Design process

#### Learn: brand personality

**Fig. 1:**
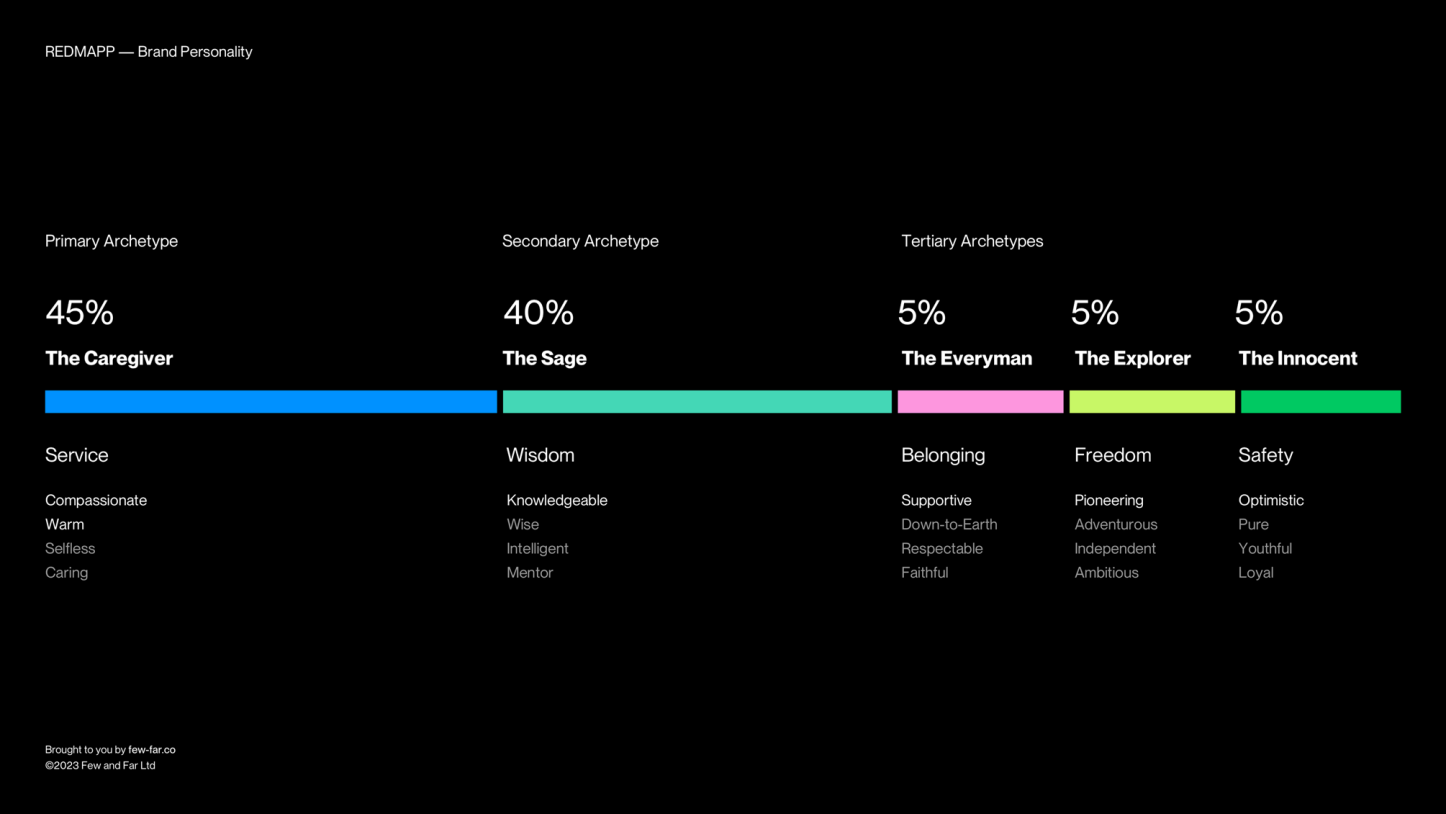
Intervention brand personality

Brand personality considered the values of the intervention, why it was important, who it was for and what the need was for the website. Addressing these values was achieved through a workshop led by the web-design team to learn from the stakeholder group what was unique to this parent population, the decision-making processes parents can endure, what this meant to them and why the website was important. Responses created the brand values for the website to be supportive, optimistic, pioneering, knowledgeable, compassionate, and warm. These led to the brand personality, the archetypes for the website (Fig. 1) which were the core tenets for the design and development phase. The purpose of the brand values and personality were to ensure these resonated with the intended parent population for the intervention thereby attracting like-minded users to a story and content which they identified with.

#### Plan: user personas

User personas represented the different parent types likely to engage with the website. Identification of user types was derived from the researcher’s (HP) clinical experience, qualitative interviews findings and discussion with the stakeholder group which included the following user personas: (1) parents of children with relapsed disease; (2) parents of children with refractory disease; (3) parents making repeated treatment decisions. Two timepoints were highlighted regarding when parents may interact with the website, when they are curious wanting to gather information and not at a treatment decision-making point and when in the process of making a treatment decision approaching the website for immediate guidance with greater anxiety or fear. Identifying these user personas supported decisions about design, navigation, and content to include which would meet the needs of all user types.

#### Plan: wireframes

Wireframes were informed by the brand values, personality, and user personas to illustrate the user journeys, what they would see and how they would navigate their way around the website based on the initial written content. The purpose was to review the wireframes based on the identified user types to see if the user journeys made sense and if anything were missing.

Feedback on the wireframes led to two revisions. The parent stories and highlighted resources content on the ‘help and resources’ page was reordered, moving the parent stories further up the page to take a more prominent position. A ‘breadcrumb’ (a secondary link for navigation) was inserted at the top of each webpage to support navigation showing the user’s location within the website.

#### Apply: digital brand foundation

**Fig. 2:**
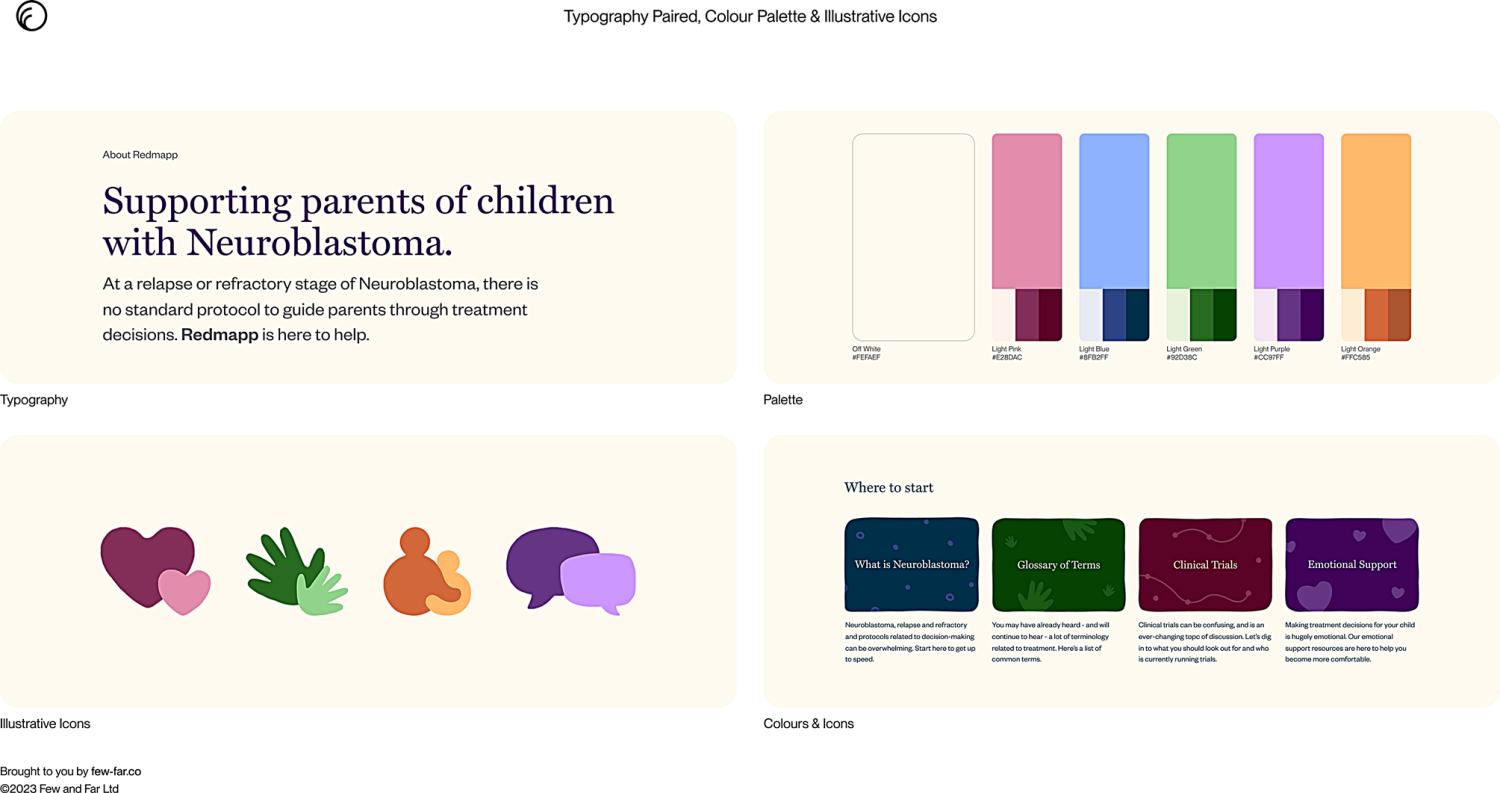
Website typology, colour palette and illustrative icons

This considered the typography, colour palate, graphics, and logo in relation to the website brand values and personality. The overall study already had a logo therefore changes to this required input and feedback from both the stakeholder and PPI group. The tone and voice of the website based on the brand values meant the study logo appeared out of place with the typography and colour palate for the website. Parent feedback suggested a new logo specifically for the website which was an output from the study so having a separate logo which aligned with this was considered acceptable and sensible. Figure 2 shows the website visuals including the typography, colour palate and graphic examples using the colour palate.

#### Apply: website designs

The web pages and user journeys defined in the wireframes were designed in full colour with illustrations. Content was inputted by the web-design team initially to be able to accurately visualise how the website would work and then subsequently updated by the researcher in the content management system. This allowed for direct access to easily update the website without future support from the web-design team, which was important for its ongoing management once developed.

The website design stage involved several iterations following feedback from the stakeholder group. Feedback related to the flow of content, breaking down information across several webpages and changes to the language given the content sensitivity in some areas for example living with cancer and considering stopping treatment. Seeing the content within the website highlighted repetition of information and how the use of signposting between webpages could support a more cohesive approach to reduce this. Once the stakeholder feedback had been actioned, the group reviewed the website again with consensus it was ready for user testing.

#### Apply: intervention user testing

Seven parents who had not been involved in the development process participated in cognitive interviews as part of user testing the intervention. Table 2 details parent characteristics of those who participated. Three parents had children who were currently receiving treatment for relapsed disease and four parents had children who had completed treatment (two for relapsed disease, one for refractory disease and one received standard front-line treatment). Cognitive interviews were completed in iterative cycles with three participants in the first cycle and two participants in the second and third cycle, totalling three rounds of interviews. Analysis involved listening to the interview recordings, summarising participants interpretations of the website relating to accessibility, acceptability, and usability, identifying areas for improvement, and making decisions on how and what to change based on user feedback. Participant interpretations were descriptive linking closely with their comments [28] to reduce the potential for misinterpretation. The website was deemed accessible with clear subject topics, short paragraphs of text with an attractive colour scheme. One mother said, *“The website is so clear*,* broken down into different subject matters which makes it more accessible.”* Parents wanted different formats of page layouts to keep their attention, increased use of sub-headings and thought referencing of other resources and incorporation of videos made the website acceptable with one mother saying, *“Having pages set-up in different formats is refreshing as you could drift off seeing the same layout all the time.”* The website appeared easy to navigate and short paragraphs meant information was easier to absorb increasing usability as captured by this father *“Short paragraphs of text are good this makes it clearer without any fancy fonts or diagrams.”*.

**Table 2:** Participant characteristics

**Parents**:	
Mothers	5
Fathers	2
**Age range**:	
22–34	
35–44 45–54 55–65	1 3 3
**Ethnicity**:	
British	7
European	
**Education**:	
Secondary College University	1 2 4

### Intervention refinement

The intervention was amended and refined following analysis of each iterative cycle of interviews, prior to starting the next round of interviews. Table 3 describes the intervention user testing amendments made following each iterative round of interviews. Following the first cycle of user testing, changes were made to increase signposting between webpages for support and easier navigation, including more parent quotes for authenticity, reformatting webpages for ease of accessibility of information and providing more information on key topics such as considering stopping treatment and looking after the whole family.

**Table 3:** User testing intervention amendments

Comments	Changes Made
***Changes made following first iterative cycle of user testing***	
Helpful to hear what other parents have said.	Parent quotes have been included in five of the webpages to provide a sense of community and help parents to feel they are not alone.
Group questions on the ‘All about clinical trials’ page.	‘All about clinical trials’ page has been reformatted with questions being broken down into three areas: clinical trials set-up, clinical trials practicalities and accessing clinical trials away from home.
Living with cancer: include information on psychological support	A link to the ‘emotional support in decision-making’ has been added to the ‘Living with cancer’ page. Signposting between webpages has increased for ease of information and to avoid duplication of the same information across the website.
Connecting with other parents: add more sub-headings as there is a lot of information.	‘Connecting with other parents’ page has been reformatted to include sub-headings for easier navigation.
Living with cancer: considering stopping treatment section does not fit within the current content.	The section ‘considering stopping treatment’ has been removed from the ‘Living with cancer’ page. There is now a separate page for this topic.
Living with cancer: include information on looking after the whole family	A section has been added titled ‘Looking after the whole family’ within the ‘Living with cancer’ page with a focus on sibling support.
***Changes made following second iterative cycle of user testing***	
Homepage: image to be more inclusive to represent children across the age ranges.	Image on the homepage changed to be more inclusive of children across the age ranges that may have relapsed or refractory neuroblastoma.
About redmapp: provide more information about the parents involved in co-design of the website.	Basic information has been added to contextualise parent experiences i.e.: parent of a child with relapsed neuroblastoma.
Emotional support in decision-making: remove considering stopping treatment information as sends a mixed message	Information on ‘Considering stopping treatment’ has been removed from the ‘Emotional support in decision-making’ page. This information is now contained within the separate page with no other signposting to this within the website.
Include information regarding the availability of clinical trials.	Clearer information and signposting have been added to the ‘All about clinical trials’ page on where information on what clinical trials are available can be found.
Emotional in decision-making: page design is bland and text heavy	Within the components of the ‘Emotional in decision-making’ page text has been broken up using colour backgrounds with additional parent quotes.
***Changes made following third iterative cycle of user testing***	
Consider different backgrounds within the boxes or thicker boarders so these stand out as individual items	Boarder of each box changes colour when the user hoovers the mouse over it to show what area is being looked at.
Homepage: increase the text size relating to the video information	Text size increased so this stands out and is more prominent on the homepage
About page: use different images across the page to break up the text	Images changed to avoid repetition
Links to other pages on the website need to be more prominent so they stand out	Links to other pages within the website have been put in bold

**Fig. 3:**
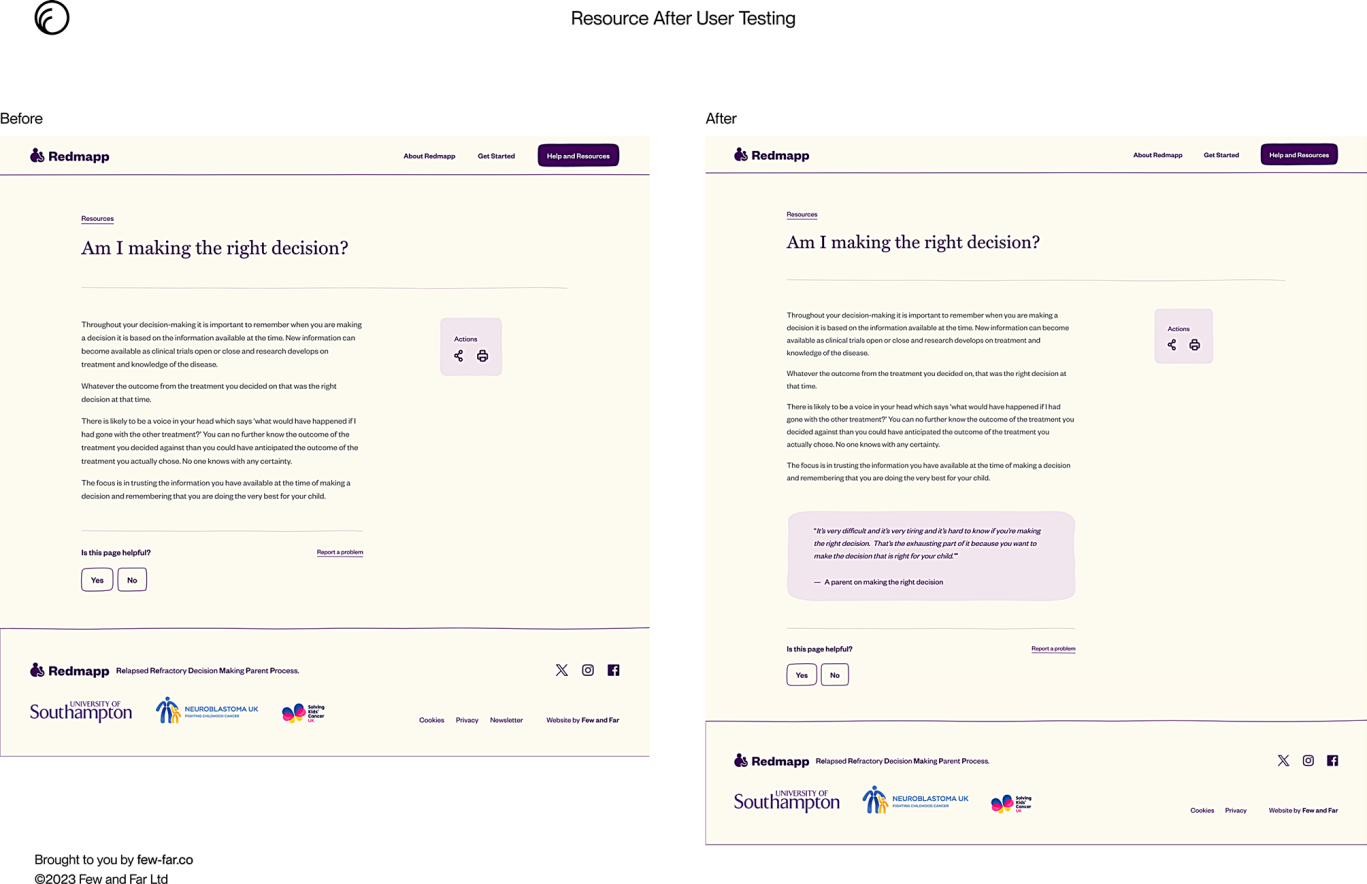
Resources webpage original and amendment after user testing

Following the second cycle of user testing changes included providing more information of the parents who were involved in the stakeholder group such as their child’s diagnosis to show credibility of the website. The ‘emotional support in decision-making’ page was considered too text heavy with the suggestion of adding parent quotes on coloured backgrounds to breakdown information. Figure 3 shows the original ‘Am I making the right decision’ webpage and the changes made to the webpage following user testing.

Following the third cycle of user testing, feedback required minimal changes. The text size was increased on the homepage and different images on the ‘About Redmapp’ page were added to avoid repetition.

## Discussion

This paper outlines the development process of a web-based intervention in the form of a decision support tool to support parents in making treatment decisions when their child has relapsed or refractory neuroblastoma. The intervention was co-designed with parents identifying user needs, iteratively developing the content and design working with a web-design company. User testing addressed whether the intervention was accessible, acceptable, and useable to optimise parent experience and engagement. Forming a one-off clinical advisory group ensured medical information was factual and correct. Involvement of healthcare professionals also supported engagement and dissemination of the intervention once developed. The benefits of co-design supported the translation of the study findings (literature review and qualitative interviews) which ensured the intervention was parent focused to meet their needs in decision-making. Study findings showed parents continued with treatment even when they recognised death was the most likely outcome for their child which is consistent with other decision-making research in children’s cancer [29]. The stakeholder group wanted to include information on considering stopping treatment, what that would mean, how their child’s trajectory would change and support at the end-of-life. Given the study findings showed this is not what parents do in practice this information was not considered at the outset. However, having information available for parents to access at a time which worked for them was an important addition recognising some parents might be thinking about this but not wanting to discuss or share with others. Having this information available provides parents with the opportunity to do their own research to prepare themselves cognitively and emotionally.

The intervention also considered how to facilitate discussions with healthcare professionals given research has shown SDM can breakdown as parents make repeated decisions. A dedicated page on ‘working with your child’s healthcare team’ incorporated videos from healthcare professionals on how medical consultants decide on treatment options, the importance of partnership working and how to request a second opinion. The downloadable ‘questions to ask your child’s consultant’ document can be used in consultations to support discussions to inform parent decision-making and facilitate a SDM approach. Having healthcare professional involvement shows the importance of such an intervention and suggests to parents that they want to work in partnership with them to make the best decisions for their child.

The development of the website took significantly longer than anticipated. Six workshops were scheduled over nine months however an additional four workshops were required. Time with the web-design team had not been factored into the stakeholder workshops partly because at the outset the researcher was unsure how this element of the work would develop and what would be required from the group for this. The development of the intervention took 18 months, double the anticipated time. Despite this all stakeholders continued to engage with the work. While this demonstrates their commitment to the work, it highlights the importance of managing expectations in co-design from the outset. In hindsight a caveat explaining the iterative nature of developing interventions with the potential need for additional workshops should have been included so parents knew what they were committing too with the need for flexibility as the process developed.

Evidence shows there is a lack of interventions which support parent decision-making [8]. Given the ever-changing treatment landscape within paediatric oncology, due to advances in research using immunotherapy and other targeted inhibitors [30, 31] in parallel with increasing use of precision medicine within cancer care [32], there is potential for this intervention to be used as a baseline and adapted for other childhood cancer or life-limiting/life-threatening illnesses where there is no standard treatment protocol and parents are making repeated treatment decisions over time.

### Future work

Further design iterations will address increasing usage of the website for other nationalities and health literacy needs. Accessibility through additional functionality, for example, the potential to translate the website into different languages, enabling the user to adjust the text size and embedding an audio function within the website are under consideration.

Another consideration was how the website would be kept updated with the integration of informal feedback as users engage with the website. This function will help keep the information relevant to parent needs in parallel with any changes in the neuroblastoma treatment landscape. The researcher will review the website three monthly as agreed with the stakeholder group to check external links and update information if processes change.

## Recommendations for intervention development using co-design

Based on our experiences of co-designing an intervention for parents for use in a sensitive subject the group devised the following recommendations to support other researchers in using this approach.

- Keep an audit trial of the intervention development process, particularly the rationale for the approach and changes made and why.
- Share a written summary each stakeholder workshop with the group. This helps ensure nothing is missed from discussions and can be used to inform the writing up of the development process.
- Audio-record group discussions to assist with writing up the summary and provides an opportunity to clarify points raised within the group.
- Co-design work is an iterative process which may require involvement beyond the initial duration set out at the start of the development process. To manage expectations with the group, ensure participants understand that a longer commitment may be needed as the work develops.
- To develop the intervention work with a design company who are recommended and/or whose portfolio is available to view. It is important to understand their approach to the intervention being developed and how this works in partnership with the co-design stakeholder group.

## Limitations

The intervention developed is for parents of children with relapsed or refractory neuroblastoma. As a result, it has limited application to other childhood cancer diagnoses in its current format. The child’s voice was not incorporated into the intervention developed however there will be some children who are of age to be involved in decision-making, with age-appropriate information to assent to the decisions made by their parents on their behalf. A lack of diversity in the parent stakeholder group and those who participated in user testing limited an inclusive and diverse perspective in the intervention developed. This could reduce relevance for some parents with using the website. Further work is required to connect with these communities, to enable the intervention to be more inclusive to meet the needs of a wider range of parents who make repeated treatment decisions over time for their terminally ill child.

## Conclusion

This is a disease specific intervention which supports parent treatment decision-making in relapsed and refractory neuroblastoma, a poor-prognosis childhood cancer. Co-design was essential to ensure the intervention met the needs of this parent population. Further work following the MRC framework will test and evaluate its impact and effectiveness in clinical practice.

## Supplementary Information

Below is the link to the electronic supplementary material.

Supplementary Material 1

## Data Availability

The datasets used and/or analysed during the current study are available from the corresponding author on reasonable request.

## Abbreviations

GDPR: General Data Protection Regulation
MRC: Medical Research Council
SDM: Shared Decision-Making
PPI: Patient and Public Involvement
UK: United Kingdom
