# Supplementary material for: Supporting parent treatment decision-making in relapsed and refractory neuroblastoma: co-design of a web-based intervention
Source: BMC Med Inform Decis Mak. 2025 Dec 8;26:12. doi: 10.1186/s12911-025-03313-z (PMC12797629; doi:10.1186/s12911-025-03313-z)
Supplement: Supplementary file 1 — Supplementary Material 1 [file 12911_2025_3313_MOESM1_ESM.docx]

**Additional file 1: Cognitive Interview Questions**

**REDMAPP Study:** **RE**lapse **D**ecision **MA**king **P**arent **P**rocess

**Researcher name**: Helen Pearson

**Accessibility**

- What do you think about the font size, style, layout, and colour of the website?
- What feeling do you get when you look at this webpage? *(ask at end of every page)*

**Acceptability**

- Is the language used appropriate and sensitive to parent needs?
- Is the language used easy to read and understand?
- Is there anything which could be confusing or misleading for parents within the website?
- Is there any content missing that should be covered within the website?

**Useability**

- Is there anything you like or do not like about the navigation of the website?
- Do you think the website offers a useful resource for parents who are making treatment decisions?
